# Vocalizations Reveal Species Differences in Endangered Lion Tamarins (Primates, Callitrichidae)

**DOI:** 10.1002/ajp.70115

**Published:** 2026-01-01

**Authors:** Maria Carolina Rodella Manzano, Ricardo J. Sawaya, Gabriela Cabral Rezende, Maria Luisa da Silva

**Affiliations:** ^1^ Graduate Program in Ecology, Institute of Biosciences Universidade de São Paulo (USP) São Paulo São Paulo Brazil; ^2^ Graduate Program in Experimental Psychology, Institute of Psychology Universidade de São Paulo (USP) São Paulo São Paulo Brazil; ^3^ IPÊ‐Institute for Ecological Research São Paulo São Paulo Brazil; ^4^ Centro de Ciências Naturais e Humanas Universidade Federal do ABC (UFABC) São Paulo São Paulo Brazil; ^5^ Institute of Biological Sciences Universidade Federal do Pará–UFPA Belém Pará Brazil

**Keywords:** acoustic communication, bioacoustics, conservation, interspecific variation, primates

## Abstract

Acoustic communication is important for social cohesion and territory defense in forest primates, including the endangered lion tamarins (genus *Leontopithecus*). Although vocalizations of individual species have been studied, there is still no comparative analysis examining whether acoustic parameters can reliably distinguish among all four species. We hypothesized that species‐specific differences in acoustic features allow discrimination among lion tamarin species, and we predicted that both spectral and temporal parameters would reveal interspecific variation. To test this, we analyzed seven shared vocalizations (long calls, whines, trills, rasps, clucks, tsicks, and peeps) from the black‐faced lion tamarin (*Leontopithecus caissara*), golden lion tamarin (*Leontopithecus rosalia*), golden‐headed lion tamarin (*Leontopithecus chrysomelas*), and black lion tamarin (*Leontopithecus chrysopygus*). Acoustic data were obtained from online sound libraries and analyzed using Raven Pro software. Spectral and temporal parameters, including frequency at 5% and 95%, peak frequency, center frequency, and bandwidth 90% were measured, followed by principal component analysis (PCA) and nonparametric statistical tests to identify species‐specific differences. Our results revealed significant interspecific differences across multiple vocalizations, with spectral parameters being the most relevant for distinguishing species, whereas temporal parameters contributed less. *L. caissara* emerged as the most acoustically distinct species, while *L. rosalia* and *L. chrysopygus* exhibited the greatest vocal similarity. In conclusion, this study provides the first comparative analysis of seven vocalization types across all four lion tamarin species, establishing an acoustic baseline, confirming the importance of spectral parameters for species differentiation, and demonstrating the potential of vocalizations for conservation applications.

## Summary

- Call types and comparison: The study analyzes and compares seven shared vocalizations across four lion tamarin species (*Leontopithecus rosalia*, *Leontopithecus chrysomelas*, *Leontopithecus chrysopygus*, and *Leontopithecus caissara*), testing the hypothesis that species‐specific acoustic parameters allow discrimination among species.
- Species‐specific differences: Significant interspecific differences were detected, driven mainly by frequency at 5% and 95%, peak frequency, and center frequency, supporting the prediction that these parameters distinguish species.
- Conservation implications: Our results provide an acoustic baseline for all four lion tamarin species across seven different vocalizations and demonstrate the potential of acoustic parameters for conservation.

## Graphical abstract

Spectral parameters of vocalizations distinguish lion tamarin species. Peak frequency, center frequency, and frequency at 5% and 95% were the most informative measures for separating species, with the trill being the vocalization that showed the most differences, and *Leontopithecus caissara* being the most distinct species of all.

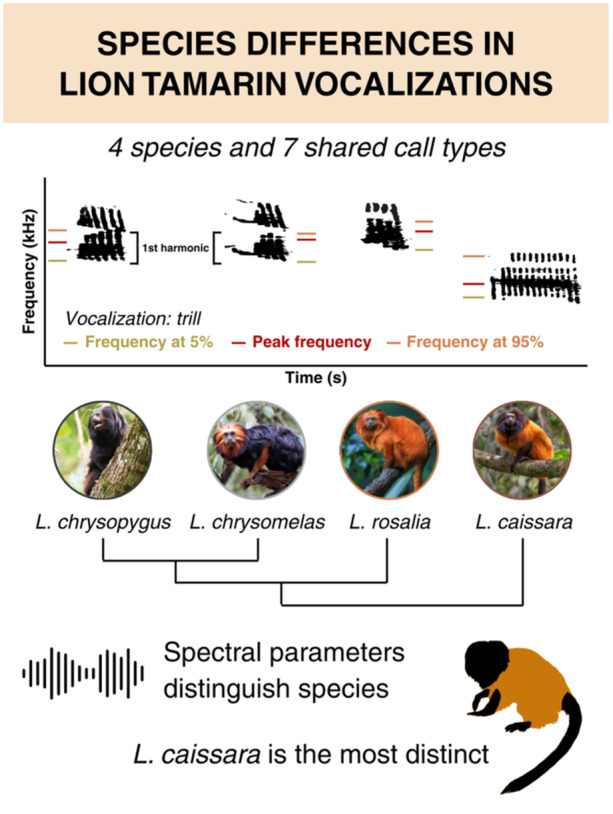

## 1 Introduction

Vocal communication has been extensively studied in various species of social primates across South America (e.g., *Alouatta caraya*: Holzmann and Areta 2020; *Alouatta pigra*: Briseño‐Jaramillo et al. 2017; *Leontopithecus rosalia*: Halloy and Kleiman 1994; Sabatini et al. 2011; *Callithrix jacchus*: Bezerra and Souto 2008; *Sapajus flavius*: Bastos et al. 2015; *Sapajus libidinosus*: Lisboa et al. 2021; *Saguinus oedipus*: Hotchkin et al. 2015), including studies on long calls, which are relevant for communication among Neotropical primates (Oliveira 2005). This type of communication plays a crucial role in maintaining group cohesion and defending territories, particularly in forested environments where other modes of communication are limited (Altmann 1967; Teixeira Da Cunha and Byrne 2009). Social group size and grooming behavior duration, for instance, are positively correlated with the extent of vocal repertoire, suggesting that species with more complex social systems have evolved more diverse vocal communication (Mccomb and Semple 2005).

Beyond vocalizations emitted in various ecological and behavioral contexts, acoustic features can indicate specific individual conditions in non‐human primates. Spectral parameters, such as fundamental, dominant, minimum, and maximum frequency values reflect the body size in different species (Hauser 1993; Hammerschmidt et al. 2000; Fitch 1997; Ey et al. 2007). Temporal parameters, such as call duration, are also related to size and age in primates (Castro and Snowdon 2000; Hammerschmidt et al. 2000; Ey et al. 2007). Furthermore, acoustic parameters of the long call in callitrichids provide important information for species identification (Mendes et al. 2009; Garbino 2015; Garbino and Martins‐Junior 2018), potentially contributing to the taxonomy of lion tamarins as previously suggested (Snowdon et al. 1986; Snowdon 1993; Garbino et al. 2014).

The genus Leontopithecus comprises four different species of lion tamarins, all considered endangered, including the black‐faced lion tamarin (Leontopithecus caissara), the golden lion tamarin (L. rosalia), the golden‐headed lion tamarin (Leontopithecus chrysomelas), and the black lion tamarin (Leontopithecus chrysopygus). The genus is distributed throughout the Atlantic Forest of Brazil, and currently, all four species have declining populations and suffer from habitat loss and fragmentation (Oliveira et al. 2020; Rezende et al. 2020; Ludwig et al. 2021; Ruiz‐Miranda et al. 2021). All these species form family social groups with a matrilinear social structure and a cooperative breeding system, in which a dominant pair reproduces while juveniles assist in infant care, and present an extensive vocal repertoire (Epple 1968; Garber 1994; Halloy and Kleiman 1994; Ruiz‐Miranda, Archer, et al. 2002).

The long call has been extensively studied in Platyrrhini primates under different approaches (Boinski et al. 1994; Kleiman and Rylands 2002; Ruiz‐Miranda, Archer, et al. 2002; Sabatini and Ruiz‐Miranda 2008; Mendes et al. 2009; Zambolli et al. 2022). In lion tamarins, the long call facilitates group cohesion, intra‐group communication, and inter‐group communication for territory defense (Snowdon 1993; Ruiz‐Miranda, Archer, et al. 2002; Sabatini and Ruiz‐Miranda 2008). Additionally, other vocalizations emitted by lion tamarins, such as the whine, have been associated with affiliative interactions within the group, food sharing, alarm calls produced in the presence of predators, and as an introductory note to the long call (Halloy and Kleiman 1994; Ruiz‐Miranda and Kleiman 2002; Lobão‐Cruz 2009).

Few studies on the acoustic communication of lion tamarins have been carried out and were primarily focused on the long call (*Leontopithecus* spp.: Snowdon et al. 1986; *L. rosalia*: Boinski et al. 1994; Halloy and Kleiman 1994; Ruiz‐Miranda, Archer, et al. 2002; Sabatini and Ruiz‐Miranda 2008; *L. chrysomelas*: Lobão‐Cruz 2009). A review of published works revealed that 17 studies focused specifically on the golden lion tamarin, representing approximately 74% of the bioacoustics knowledge for the genus. Only one master's thesis addressed *L. chrysomelas* (Lobão‐Cruz), while *L. chrysopygus* was investigated mainly in the context of passive acoustic monitoring (Zambolli et al. 2022; Manzano et al. 2024), and no data were available for *L. caissara*, except for an abstract in conference proceedings (Garbino et al. 2014). The most significant differences among species within the genus are associated with maximum frequency, number of syllables, and duration of the long call (Garbino et al. 2014; Zambolli 2017, unpublished data).

In this context, our study compared different vocalizations among the four species of lion tamarins (*Leontopithecus*) to investigate whether acoustic parameters can effectively distinguish them. We hypothesize that there are acoustic differences among species not only in the long call, as previously reported for three species, but also across other types of vocalizations that have been less studied. We predict that both spectral and temporal parameters will reveal interspecific variation, allowing the identification of species‐specific acoustic signatures. Comparing the acoustic diversity among species within the genus *Leontopithecus* is the first step toward understanding how evolutionary, morphological, and ecological factors shape acoustic communication and toward establishing a baseline for future studies on intraspecific variation and individual recognition, which are key aspects of behavioral and conservation research.

## 2 Materials and Methods

This research adhered to the American Society of Primatologists (ASP) Principles for the Ethical Treatment of Non‐Human Primates and to the legal requirements of Brazil. Permits were duly provided by the Chico Mendes Institute for Biodiversity Conservation (ICMBio, Brazil: SISBIO 78701‐4).

### 2.1 Data Collection

Acoustic data for the four lion tamarin species (*Leontopithecus*) were provided by the Museu de Diversidade Biológica (MDBio) Audiovisual Collection, in the University of Campinas (UNICAMP, São Paulo, Brazil), integrated by the Fonoteca Neotropical Jacques Vielliard (FNJV). Nineteen recordings were obtained, distributed among the species as follows: *L. caissara* (*N* = 4), *L. rosalia* (*N* = 2), *L. chrysomelas* (*N* = 7), *L. chrysopygus* (*N* = 6). The recordings mostly comprise long calls and whine vocalizations. Another 16 recordings were made available from the Macaulay Library at the Cornell Lab of Ornithology, including *L. rosalia* (*N* = 2), *L. chrysomelas* (*N* = 6), and *L. chrysopygus* (*N* = 8). These recordings were originally collected from both wild and captive individuals, with metadata sometimes lacking detailed field information (see Supporting Information SI). All files were provided in uncompressed WAV format, with sampling rates ≥ 44.1 kHz, ensuring adequate frequency resolution. Only high‐quality vocalizations were selected for analysis with clear temporal and spectral parameters visible in both oscillograms and spectrograms. We analyzed seven vocalizations common to the four species of the genus, including long calls, whines, trills, rasps, clucks, tsicks, and peeps.

### 2.2 Data Analysis

Spectral parameters, including low frequency (Hz), high frequency (Hz), frequency at 5% (Hz), frequency at 95% (Hz), peak frequency (Hz), center frequency (Hz), and bandwidth containing 90% of the song's energy (Hz), were estimated through spectrogram analysis (FFT = 1024, 90% overlap, “Hann” window). Temporal parameters, including delta time (s) and duration at 90% (s), were measured through the oscillogram (Table 1). The recordings were analyzed using Raven Pro software (v1.6, K. Lisa Yang Center for Conservation Bioacoustics, Cornell Lab of Ornithology, Cornell University 2022). The choice of parameters followed previous studies on lion tamarins, where minimum, maximum, and peak frequencies are commonly used (e.g., Halloy and Kleiman 1994; Ruiz‐Miranda, Archer, et al. 2002; Sabatini and Ruiz‐Miranda 2008). To reduce observer bias, we also included the 5% and 95% frequencies, which are automatically calculated from the energy distribution of the signal and provide more objective and comparable estimates. The spectrogram settings (FFT = 1024, Hann window, and high overlap) increase spectral resolution and minimize loss of information between time windows, ensuring more precise frequency measurements (Fischer et al. 2013). We acknowledge that visually selecting signals from the spectrogram may introduce observer bias; however, the inclusion of automatically calculated parameters (frequency at 5% and 95%, and BW90) helps reduce this limitation and provides more objective measurements.

**Table 1:** Description of acoustic parameters measured from four species of lion tamarins (*Leontopithecus*) vocalizations (Charif et al. 2010).

Acoustic parameter	Type	Unit	Description
Low frequency (low freq)	Spectral	Hz	The lower frequency bound of the selection.
High Frequency (high freq)	Spectral	Hz	The upper frequency bound of the selection.
Frequency at 5% (Freq. 5%)	Spectral	Hz	Frequency below which 5% of the call's energy is contained.
Frequency at 95% (Freq. 95%)	Spectral	Hz	Frequency below which 95% of the call's energy is contained.
Peak frequency (peak freq)	Spectral	Hz	The frequency with the highest energy in the call.
Center frequency (center freq)	Spectral	Hz	The frequency that divides the call into two intervals with equal energy.
Bandwidth 90% (BW90)	Spectral	Hz	The difference between the 5% and 95% frequencies.
Delta time	Temporal	s	The difference between the beginning time and the end time for the selection.
Duration 90% (Dur90)	Temporal	s	The difference between the 5% and 95% times.

Abbreviations: Hz = hertz, s = seconds.

We tested the data for normality and homoscedasticity to determine their suitability for parametric ANOVA tests. Since the data did not meet such assumptions, we opted for nonparametric tests for subsequent analyses. We performed Kruskal−Wallis tests for each spectral and temporal variable, comparing different species for each type of vocalization. The Kruskal−Wallis tests were performed using the “*kruskal*.*test*” function in “*stats*” package in R (R Core Team 2020). When these tests indicated significant differences (*p* < 0.05), we performed post hoc comparisons using the Wilcoxon test to identify which pairs of species showed significant differences, using the “*wilcox*.*test*” function.

We performed multivariate analyses using principal component analysis (PCA) and cluster analysis to identify groups based on all seven types of vocalizations of the four species, considering the following spectral parameters: frequency 5% (Hz), frequency 95% (Hz), peak frequency (Hz), center frequency (Hz), and bandwidth containing 90% of the song's energy (Hz). The PCA was implemented using the “*prcomp*” function from the “*stats*” package in R (R Core Team 2020). The resulting PCA scatter plots and cluster analysis were generated using the “*ggplot2*” package (Wickham 2016). We also conducted a hierarchical clustering analysis to explore acoustic similarity patterns among the four species, using the “*hclust*” function from the “*stats*” package in R (R Core Team 2020). The Euclidean distance matrix was computed from the mean values of the acoustic parameters for each species based on all seven types of vocalizations, and we employed hierarchical clustering using the complete linkage method to group the species.

## 3 Results

**Figure 1:**
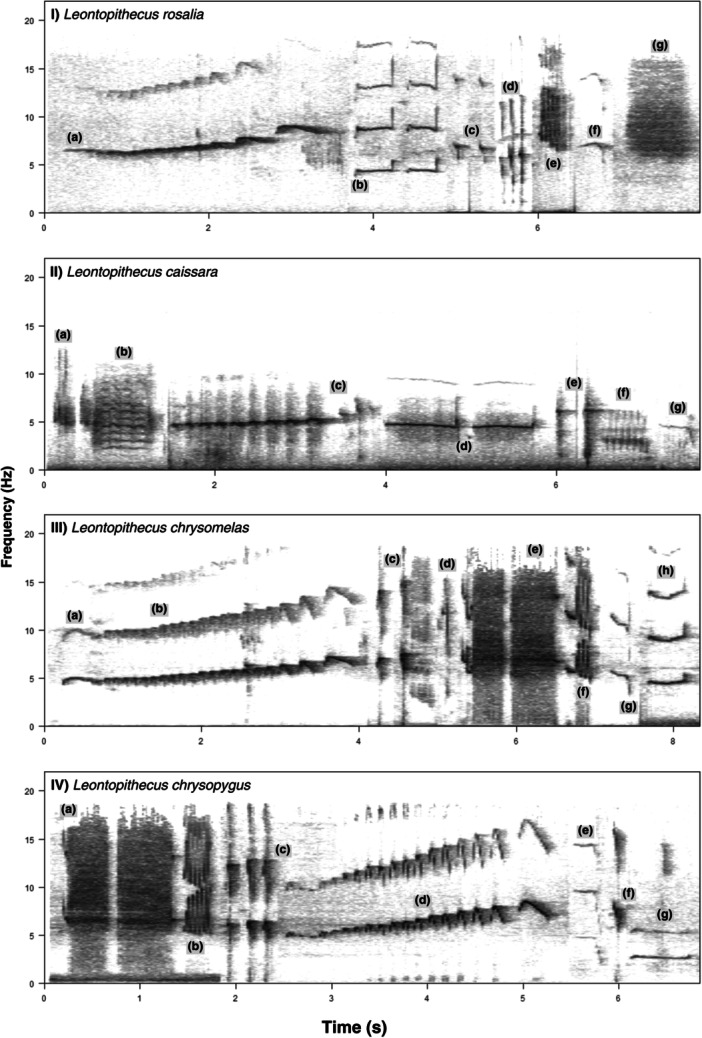
Sonograms of vocalizations emitted by (I) *Leontopithecus rosalia*: (a) two‐syllable long call, (b) two whines, (c) two clucks (ML90226), (d) three tsicks (FNJV00032606), (e) trill, (f) peep, and (g) rasp (ML90219). (II) *Leontopithecus caissara*: (a) two clucks, (b) trill‐rasp (FNJV0007942), (c) two‐phrases long call (FNJV0044354), (d) two whines, (e) two tsicks (FNJV0044355), (f) trill, and (g) peep (FNJV0044353). (III) *Leontopithecus chrysomelas*: (a) introductory note of the long call, (b) two‐phrases long call, (c) two tsicks, (d) cluck, (e) trill‐rasp‐rasp, (f) trill, (g) peep (ML90211), and (h) whine (ML90212). (IV) *Leontopithecus chrysopygus*: (a) two rasps, (b) trill, (c) three tsicks, (d) two‐phrases long call (ML90217), (e) peep, (f) cluck, and (g) whine (ML90218). The recordings were available from Macaulay Library (ML) and Fonoteca Neotropical Jaqques Viellard (FNJV). The figures were created using “seewave” and “tuneR” packages from R (R Core Team 2020), using FFT = 1024, 90% overlap, “Hann” window.

We considered seven different types of vocalizations emitted by species of the genus *Leontopithecus*, including long calls, whines, trills, rasps, clucks, tsicks, and peeps. The vocalizations were variable in terms of frequency modulation and duration (e.g., with both longer and shorter trills) and could be used in different ways (Figure 1, Table 2). Some works separate the golden lion tamarin long call into different phrases, used in different behavioral contexts (Halloy and Kleiman 1994; Ruiz‐Miranda and Kleiman 2002; Sabatini and Ruiz‐Miranda 2010; Sabatini et al. 2011). The first phrase is used for group coordination when moving, the second for group cohesion when an individual gets lost, and the third is mainly used to demarcate territory and intergroup spacing (Ruiz‐Miranda and Kleiman 2002; Sabatini and Ruiz‐Miranda 2010; Sabatini et al. 2011). In this work, we considered the 2‐phrase long calls for analysis, excluding long calls with final notes used in the context of territorial defense.

**Table 2:** Acoustic parameters of the seven different vocalizations of four species of lion tamarins (*Leontopithecus*) including median and standard deviation values.

*Leontopithecus caissara*							
Acoustic parameters	2‐phrase long call (13)	Whine (20)	Peep (6)	Trill (30)	Tsick (44)	Cluck (10)	Rasp (14)
Low freq (Hz)	4647.4 ± 1111.3	2957.1 ± 731.5	2299.2 ± 987.5	3424.2 ± 977.3	1216.6 ± 452.8	3991.1 ± 595.2	1308.9 ± 322.4
High freq (Hz)	9235 ± 1471.2	7659 ± 548.1	5047.3 ± 631	7465.4 ± 886	6720.9 ± 326.6	8405.2 ± 1405.9	10341 ± 869.8
Freq. 5% (Hz)	5296.9 ± 933.1	4453.1 ± 202.3	2437.5 ± 990.4	4242.2 ± 1211.9	1359.4 ± 578.4	5062.5 ± 218.8	1781.3 ± 310.7
Freq. 95% (Hz)	6187.5 ± 1241.8	4687.5 ± 238.1	4757.8 ± 597.9	6023.4 ± 1012.7	6281.3 ± 1054	6867.2 ± 835.7	6093.8 ± 187.1
Peak freq (Hz)	5765.6 ± 815.4	4546.9 ± 268.4	4429.7 ± 927.8	4640.6 ± 1256	1593.8 ± 1567.8	5507.8 ± 696.7	4734.4 ± 1125.9
Center freq (Hz)	5718.8 ± 962.3	4546.9 ± 248.6	3750 ± 892.4	4968.8 ± 1171.1	2296.9 ± 1316.3	5742.2 ± 480.7	4664.1 ± 171
Bw90 (Hz)	1125 ± 343.7	281.3 ± 106	2132.8 ± 554.4	1921.9 ± 559.6	4804.7 ± 1047.5	1945.3 ± 737.0	4312.5 ± 251.4
Delta time (s)	2.911 ± 0.602	0.748 ± 0.147	0.439 ± 0.096	0.278 ± 0.259	0.075 ± 0.01	0.076 ± 0.029	0.791 ± 0.161
Dur90 (s)	1.832 ± 0.536	0.616 ± 0.133	0.366 ± 0.064	0.226 ± 0.228	0.047 ± 0.01	0.031 ± 0.030	0.693 ± 0.141

*Leontopithecus chrysomelas*							
Acoustic parameters	2‐phrase long call (58)	Whine (25)	Peep (42)	Trill (45)	Tsick (37)	Cluck (40)	Rasp (18)
Low freq (Hz)	5386.5 ± 845.9	4571.4 ± 1521.7	3252.0 ± 1011.4	2297.7 ± 1042.5	1156.6 ± 1065.7	3084.3 ± 1286.0	1185.3 ± 983.9
High freq (Hz)	9474.0 ± 1519.4	6838.5 ± 1178.3	7125.9 ± 684.3	9235.5 ± 1727.2	6697.4 ± 1306.2	6988.0 ± 1336.6	17,631.1 ± 3239.7
Freq. 5% (Hz)	6460.0 ± 941.9	5203.1 ± 1582.7	4557.9 ± 1256.5	4781.3 ± 1275.7	4220.5 ± 1480.5	5250.0 ± 729.2	4546.9 ± 1068.9
Freq. 95% (Hz)	8591.7 ± 1320.5	5718.8 ± 1250.4	6869.1 ± 620.1	8203.1 ± 1223.3	6000.0 ± 1209.5	6375.0 ± 1355.8	10,921.9 ± 1048.1
Peak freq (Hz)	7472.0 ± 1211.2	5343.8 ± 1545.2	6804.5 ± 1112.3	7312.5 ± 1083.0	5625.0 ± 1259.0	6281.3 ± 961.7	5156.3 ± 1493.5
Center freq (Hz)	7278.2 ± 1053.5	5390.6 ± 1638.5	6718.4 ± 994.0	7031.3 ± 1011.1	5531.3 ± 1206.1	6234.4 ± 955.8	6539.1 ± 1140.5
Bw90 (Hz)	2239.5 ± 702.0	515.6 ± 572.4	2390.2 ± 1278.4	3937.5 ± 1011.8	2670.1 ± 1624.0	1171.9 ± 899.7	7312.5 ± 1128.0
Delta time (s)	2.870 ± 1.066	0.894 ± 0.289	0.248 ± 0.033	0.268 ± 0.069	0.075 ± 0.014	0.083 ± 0.048	0.662 ± 0.316
Dur90 (s)	2.133 ± 0.777	0.652 ± 0.194	0.193 ± 0.029	0.181 ± 0.064	0.033 ± 0.008	0.046 ± 0.027	0.520 ± 0.238

*Leontopithecus chrysopygus*							
Acoustic parameters	2‐phrase long call (59)	Whine (30)	Peep (9)	Trill (71)	Tsick (21)	Cluck (10)	Rasp (46)
Low freq (Hz)	5055.1 ± 622.2	2605.8 ± 698.6	2205 ± 681.8	4039.1 ± 450.9	2185.6 ± 1129.4	4973.7 ± 264.8	3751.3 ± 958.6
High freq (Hz)	9888.9 ± 1210.4	5853.2 ± 1135.1	5659.5 ± 1052.2	10,003.6 ± 1212.4	7071.1 ± 721.2	9114 ± 1121.7	16,693.2 ± 2645.6
Freq. 5% (Hz)	6093.7 ± 510.7	3402.2 ± 989.9	3660.6 ± 821.7	5340.2 ± 289.3	5598.6 ± 775.5	6223.1 ± 290.3	5426.4 ± 296.7
Freq. 95% (Hz)	8613.3 ± 769.8	4429.7 ± 1220.7	5512.5 ± 976.3	9302.3 ± 1120.3	6804.5 ± 690.1	7622.8 ± 952.4	11,692.5 ± 1712.2
Peak freq (Hz)	7078.1 ± 827.5	3680.0 ± 1188.7	5254.1 ± 772.4	6976.8 ± 1549.5	6718.4 ± 651,9	7170.6 ± 553.7	6459.96 ± 1844.96
Center freq (Hz)	7149 ± 619.4	3636.9 ± 1066.3	5168 ± 479.6	6890.6 ± 599.2	6675.3 ± 658.3	7084.4 ± 405.5	6976.8 ± 664.8
Bw90 (Hz)	2411.7 ± 549.3	775.2 ± 688.4	2325.6 ± 498.5	4048.2 ± 1018.4	1033.6 ± 584.6	1162.8 ± 942	6115.4 ± 1599.6
Delta time (s)	3.42 ± 0.803	0.698 ± 0.239	0.341 ± 0.057	0.393 ± 0.115	0.102 ± 0.016	0.105 ± 0.018	0.544 ± 0.125
Dur90 (s)	2.386 ± 0.706	0.561 ± 0.225	0.2723 ± 0.048	0.315 ± 0.098	0.047 ± 0.013	0.042 ± 0.014	0.455 ± 0.111

*Leontopithecus rosalia*							
Acoustic parameters	2‐phrase long call (30)	Whine (47)	Peep (13)	Trill (42)	Tsick (30)	Cluck (16)	Rasp (8)
Low freq (Hz)	4812.7 ± 801.8	3140.4 ± 357.9	4056.3 ± 856.8	3112.8 ± 1108.4	2672.1 ± 1302.4	5592.1 ± 702.1	2870.2 ± 1003.2
High freq (Hz)	8529.7 ± 1334.2	6120.7 ± 929.4	6829.4 ± 802.5	8461.2 ± 2400.7	6817.3 ± 790.1	9310.9 ± 941.7	16,810.8 ± 1476.6
Freq. 5% (Hz)	5835.5 ± 773.6	3919.0 ± 362.6	4522 ± 1006.8	4909.6 ± 1062.0	5232.4 ± 1488.6	6753.8 ± 940.7	5835.5 ± 253.7
Freq. 95% (Hz)	7816.6 ± 1113.8	5081.8 ± 1104.1	6718.4 ± 809.1	7278.2 ± 2043.5	6530.9 ± 790.2	8957.8 ± 737.8	9948.3 ± 1373
Peak freq (Hz)	6739.9 ± 1061.8	4171.9 ± 751.3	6703.1 ± 874.6	6115.4 ± 963.5	6234.4 ± 779.9	8268.8 ± 1079.6	6373.8 ± 1116
Center freq (Hz)	6739.9 ± 887.7	4134.4 ± 370.9	6656.3 ± 893.2	6115.4 ± 1230	6117.2 ± 807.8	8441 ± 875.3	6804.5 ± 804.2
Bw90 (Hz)	1938 ± 921.8	1119.7 ± 975.5	1968.8 ± 626.7	2983.9 ± 1288.6	1205.9 ± 1174.2	2325.6 ± 639.6	4134.4 ± 1173.1
Delta time (s)	3.062 ± 0.772	0.599 ± 0.204	0.325 ± 0.063	0.358 ± 0.148	0.087 ± 0.027	0.154 ± 0.048	0.55 ± 0.106
Dur90 (s)	2.249 ± 0.621	0.488 ± 0.159	0.232 ± 0.053	0.275 ± 0.129	0.037 ± 0.017	0.102 ± 0.036	0.429 ± 0.079

*Note:* Number in parentheses refers to the number of vocalizations analyzed for each vocalization type.

Abbreviations: BW90 = bandwidth 90%, Center freq = center frequency, Delta time = time difference between start and end of the call, Dur90 = duration containing 90% of the energy, Freq. 5% = frequency at 5% of the energy, Freq. 95% = frequency at 95% of the energy, High freq = high frequency, Hz = hertz, Low freq = low frequency, Peak freq = peak frequency, s = seconds.

The Kruskal−Wallis rank sum test was performed to assess differences in all spectral and temporal parameters among species, evaluating all vocalizations of the genus for identifying possible interspecies differences. The species that differed most from the others in terms of the different types of vocalizations was *L. caissara*, in Trill, Tsick, Peep, and Rasp, followed by *L. rosalia* in Whine and Cluck. Trill and Cluck were the vocalizations that presented the greatest number of differences in paired comparisons among those analyzed, being relevant to separate species in 76% of the matchups.

For long calls (mainly 2‐phrase long calls), significant differences were observed in spectral and temporal parameters (Table 3). In paired comparisons between species, *L. chrysopygus* differed from the other three lion tamarins in only one acoustic parameter (delta time), in three more parameters from *L. chrysomelas* (frequency at 5%, BW90, and duration 90%), and in one more parameter from *L. rosalia* (frequency at 95%). *L. caissara* differed from *L. chrysopygus* in all parameters, except frequency at 5%. Besides that, *L. caissara* differed from *L. chrysomelas* and *L. rosalia* in almost all parameters, except temporal ones. Similarly, *L. rosalia* differed from *L. chrysomelas* in all parameters, except BW90.

**Table 3:** Results of the pairwise comparisons (*p* value of Wilcoxon post hoc tests) for all acoustic parameters of the seven types of vocalizations emitted by the four species of the genus *Leontopithecus*.

Acoustic parameters	Species	*L. caissara*	*L. rosalia*	*L. chrysomelas*
**Vocalization: Long call**				
Freq. 5% (Hz)	*L. rosalia*	0.8015		
	*L. chrysomelas*	**0.0144**	**< 0.001**	
	*L. chrysopygus*	0.2414	0.0723	**0.0043**
Freq. 95% (Hz)	*L. rosalia*	**0.0160**		
	*L. chrysomelas*	**< 0.001**	**0.0346**	
	*L. chrysopygus*	**< 0.001**	**0.0217**	0.6706
Peak freq (Hz)	*L. rosalia*	**0.0073**		
	*L. chrysomelas*	**< 0.001**	**0.0183**	
	*L. chrysopygus*	**< 0.001**	0.2798	0.0963
Center freq (Hz)	*L. rosalia*	**0.029**		
	*L. chrysomelas*	**0.0002**	**0.0095**	
	*L. chrysopygus*	**0.0003**	0.0545	0.2108
Bw90 (Hz)	*L. rosalia*	**< 0.001**		
	*L. chrysomelas*	**< 0.001**	0.7613	
	*L. chrysopygus*	**< 0.001**	0.0567	**0.0363**
Delta time (s)	*L. rosalia*	0.6476		
	*L. chrysomelas*	0.738	0.3716	
	*L. chrysopygus*	**0.0481**	**0.0401**	**0.0027**
Dur90 (s)	*L. rosalia*	0.3213		
	*L. chrysomelas*	0.6718	0.5642	
	*L. chrysopygus*	**0.0449**	0.1813	**0.0239**
**Vocalization: Whine**				
Freq. 5% (Hz)	*L. rosalia*	**< 0.001**		
	*L. chrysomelas*	0.689	**0.0163**	
	*L. chrysopygus*	**< 0.001**	**< 0.001**	**< 0.001**
Freq. 95% (Hz)	*L. rosalia*	0.166		
	*L. chrysomelas*	**< 0.001**	**0.0148**	
	*L. chrysopygus*	0.3992	**0.0133**	**< 0.001**
Peak freq (Hz)	*L. rosalia*	0.0524		
	*L. chrysomelas*	0.2045	**0.0246**	
	*L. chrysopygus*	**< 0.001**	**< 0.001**	**< 0.001**
Center freq (Hz)	*L. rosalia*	**< 0.001**		
	*L. chrysomelas*	0.5447	**< 0.001**	
	*L. chrysopygus*	**< 0.001**	**< 0.001**	**< 0.001**
Bw90 (Hz)	*L. rosalia*	**< 0.001**		
	*L. chrysomelas*	**< 0,001**	0.2583	
	*L. chrysopygus*	**< 0.001**	0.6232	0.3977
Delta time (s)	*L. rosalia*	**< 0.001**		
	*L. chrysomelas*	0.233	**< 0.001**	
	*L. chrysopygus*	0.1349	0.0527	0.0583
Dur90 (s)	*L. rosalia*	**< 0.001**		
	*L. chrysomelas*	0.7233	**< 0.001**	
	*L. chrysopygus*	0.067	0.0637	0.2301
**Vocalization: Trill**				
Freq. 5% (Hz)	*L. rosalia*	**< 0.001**		
	*L. chrysomelas*	**0.0068**	0.1809	
	*L. chrysopygus*	**< 0.001**	**0.0030**	**< 0.001**
Freq. 95% (Hz)	*L. rosalia*	**< 0.001**		
	*L. chrysomelas*	**< 0.001**	**0.0249**	
	*L. chrysopygus*	**< 0.001**	**< 0.001**	**< 0.001**
Peak freq (Hz)	*L. rosalia*	**< 0.001**		
	*L. chrysomelas*	**< 0.001**	**< 0.001**	
	*L. chrysopygus*	**< 0.001**	**0.0024**	0.5481
Center freq (Hz)	*L. rosalia*	**< 0.001**		
	*L. chrysomelas*	**< 0.001**	**0.0137**	
	*L. chrysopygus*	**< 0.001**	**< 0.001**	0.7467
Bw90 (Hz)	*L. rosalia*	**< 0.001**		
	*L. chrysomelas*	**< 0.001**	**< 0.001**	
	*L. chrysopygus*	**< 0.001**	**< 0.001**	0.5001
Delta time (s)	*L. rosalia*	0.1245		
	*L. chrysomelas*	0.5924	**< 0.001**	
	*L. chrysopygus*	**0.0071**	0.1414	**< 0.001**
Dur90 (s)	*L. rosalia*	0.3489		
	*L. chrysomelas*	0.1581	**< 0.001**	
	*L. chrysopygus*	**0.0099**	0.0677	**< 0.001**
**Vocalization: Tsick**				
Freq. 5% (Hz)	*L. rosalia*	**< 0.001**		
	*L. chrysomelas*	**< 0.001**	**< 0.001**	
	*L. chrysopygus*	**< 0.001**	0.1329	**< 0.001**
Freq. 95% (Hz)	*L. rosalia*	**0.0204**		
	*L. chrysomelas*	0.1763	0.3773	
	*L. chrysopygus*	**< 0.001**	0.3057	0.1109
Peak freq (Hz)	*L. rosalia*	**< 0.001**		
	*L. chrysomelas*	**< 0.001**	**0.0109**	
	*L. chrysopygus*	**< 0.001**	0.0798	**< 0.001**
Center freq (Hz)	*L. rosalia*	**< 0.001**		
	*L. chrysomelas*	**< 0.001**	**0.0127**	
	*L. chrysopygus*	**< 0.001**	**0.0831**	**< 0.001**
Bw90 (Hz)	*L. rosalia*	**< 0.001**		
	*L. chrysomelas*	**< 0.001**	**0.0023**	
	*L. chrysopygus*	**< 0.001**	0.3148	**< 0.001**
Delta time (s)	*L. rosalia*	**0.0493**		
	*L. chrysomelas*	0.9849	0.1208	
	*L. chrysopygus*	**< 0.001**	**0.0029**	**< 0.001**
Dur90 (s)	*L. rosalia*	**0.002**		
	*L. chrysomelas*	**< 0.001**	0.1409	
	*L. chrysopygus*	0.9776	**0.0037**	**< 0.001**
**Vocalization: Cluck**				
Freq. 5% (Hz)	*L. rosalia*	**< 0.001**		
	*L. chrysomelas*	**0.0222**	**< 0.001**	
	*L. chrysopygus*	**0.0002**	**0.0188**	**0.0063**
Freq. 95% (Hz)	*L. rosalia*	**< 0.001**		
	*L. chrysomelas*	0.0616	**< 0.001**	
	*L. chrysopygus*	0.063	**0.0007**	**0.0053**
Peak freq (Hz)	*L. rosalia*	**< 0.001**		
	*L. chrysomelas*	**0.0013**	**< 0.001**	
	*L. chrysopygus*	**< 0.001**	**0.0234**	**0.0174**
Center freq (Hz)	*L. rosalia*	**< 0.001**		
	*L. chrysomelas*	**0.0104**	**< 0.001**	
	*L. chrysopygus*	**0.0008**	**0.0031**	**0.0138**
Bw90 (Hz)	*L. rosalia*	0.6162		
	*L. chrysomelas*	**< 0.001**	**< 0.001**	
	*L. chrysopygus*	**0.0311**	0.0507	0.4589
Delta time (s)	*L. rosalia*	**< 0.001**		
	*L. chrysomelas*	0.1782	**< 0.001**	
	*L. chrysopygus*	**0.0373**	**0.0014**	0.0918
Dur90 (s)	*L. rosalia*	**0.002**		
	*L. chrysomelas*	0.112	**< 0.001**	
	*L. chrysopygus*	0.3438	**< 0.001**	1.00
**Vocalization: Peep**				
Freq. 5% (Hz)	*L. rosalia*	**0.0096**		
	*L. chrysomelas*	**0.0126**	0.526	
	*L. chrysopygus*	0.1404	**0.0275**	0.0905
Freq. 95% (Hz)	*L. rosalia*	**0.0033**		
	*L. chrysomelas*	**< 0.001**	0.0584	
	*L. chrysopygus*	**0.039**	0.1087	**< 0.001**
Peak freq (Hz)	*L. rosalia*	**0.0032**		
	*L. chrysomelas*	**< 0.001**	0.1906	
	*L. chrysopygus*	0.14	**0.0121**	**< 0.001**
Center freq (Hz)	*L. rosalia*	**0.0033**		
	*L. chrysomelas*	**< 0.001**	0.1809	
	*L. chrysopygus*	**0.0386**	**0.023**	**< 0.001**
Bw90 (Hz)	*L. rosalia*	0.4558		
	*L. chrysomelas*	0.2751	**0.0245**	
	*L. chrysopygus*	0.8639	0.2162	0.3289
Delta time (s)	*L. rosalia*	**0.0014**		
	*L. chrysomelas*	**< 0.001**	**< 0.001**	
	*L. chrysopygus*	**0.0256**	0.2624	**< 0.001**
Dur90 (s)	*L. rosalia*	**0.0057**		
	*L. chrysomelas*	**< 0.001**	**< 0.001**	
	*L. chrysopygus*	0.0872	**0.0488**	**< 0.001**
**Vocalization: Rasp**				
Freq. 5% (Hz)	*L. rosalia*	**< 0.001**		
	*L. chrysomelas*	**< 0.001**	**0.0001**	
	*L. chrysopygus*	**< 0.001**	**0.0155**	**< 0.001**
Freq. 95% (Hz)	*L. rosalia*	**< 0.001**		
	*L. chrysomelas*	**< 0.001**	**0.016**	
	*L. chrysopygus*	**< 0.001**	**0.0341**	0.7709
Peak freq (Hz)	*L. rosalia*	**< 0.001**		
	*L. chrysomelas*	**0.0082**	0.0024	
	*L. chrysopygus*	**< 0.001**	**0.8741**	**< 0.001**
Center freq (Hz)	*L. rosalia*	**< 0.001**		
	*L. chrysomelas*	**< 0.001**	0.3309	
	*L. chrysopygus*	**< 0.001**	0.9903	0.2234
Bw90 (Hz)	*L. rosalia*	0.5605		
	*L. chrysomelas*	**< 0.001**	**< 0.001**	
	*L. chrysopygus*	**< 0.001**	**0.0085**	**0.0092**
Delta time (s)	*L. rosalia*	**< 0.001**		
	*L. chrysomelas*	0.1929	0.2379	
	*L. chrysopygus*	**< 0.001**	0.8742	0.0569
Dur90 (s)	*L. rosalia*	**< 0.001**		
	*L. chrysomelas*	0.0805	0.1408	
	*L. chrysopygus*	**< 0.001**	0.6349	0.063

*Note:* Significant *p* values (< 0.05) are highlighted in bold.

Abbreviations: BW90 = bandwidth 90%, Center freq = center frequency, Delta time = time difference between start and end of the call, Dur90 = duration containing 90% of the energy, Freq. 5% = frequency at 5% of the energy, Freq. 95% = frequency at 95% of the energy, High freq = high frequency, Hz = hertz, Low freq = low frequency, Peak freq = peak frequency, s = seconds.

In long calls, the spectral parameters frequency at 95%, peak frequency, center frequency, and BW90 were the most relevant to separate the species, presenting differences in at least four of the six paired comparisons. Meanwhile, temporal parameters were only relevant to separate *L. chrysopygus* from the three other species. The species that differed most from the others was *L. caissara*, presenting significance in six of the seven parameters in comparison with *L. chrysopygus*, and five of the seven parameters in comparison with *L. chrysomelas*. Next, *L. rosalia* separated from *L. caissara* and *L. chrysomelas* by four of the seven parameters, all spectral. *L. chrysomelas* and *L. chrysopygus* were separated by four of the seven parameters. The species that showed the least differences were *L. rosalia* and *L. chrysopygus*.

For the other vocalizations (whine, trill, tsick, cluck, peep, and rasp), interspecific differences were also observed, particularly in spectral parameters. For whine, trill, and tsick, spectral parameters (frequency at 5%, peak frequency, center frequency, BW90) showed significant differences between almost all species. *L. caissara* differed most consistently from the other species, especially in trill, tsick, peep, and rasp, showing significant differences in almost all spectral parameters in paired comparisons. *L. rosalia* differed mainly in whine and cluck, with significant differences in frequency at 5%, peak, and center frequency. *L. chrysopygus* was often distinguished from the other species by delta time and duration 90% in tsick and trill, although spectral parameters also contributed in some vocalizations. *L. chrysomelas* showed intermediate differentiation, differing from *L. chrysopygus* and *L. rosalia* in several spectral parameters (frequency at 5%, peak, center, BW90), but less consistently than *L. caissara*.

We observed that even when considering all the different types of vocalizations emitted by lion tamarins, temporal parameters appear to be less relevant to differentiate species than spectral parameters. Of all comparisons considering the seven types of vocalizations, the parameters that appeared most important to differentiate species were frequency at 5% (30), center frequency (29), peak frequency (28), and frequency at 95% (25).

**Figure 2:**
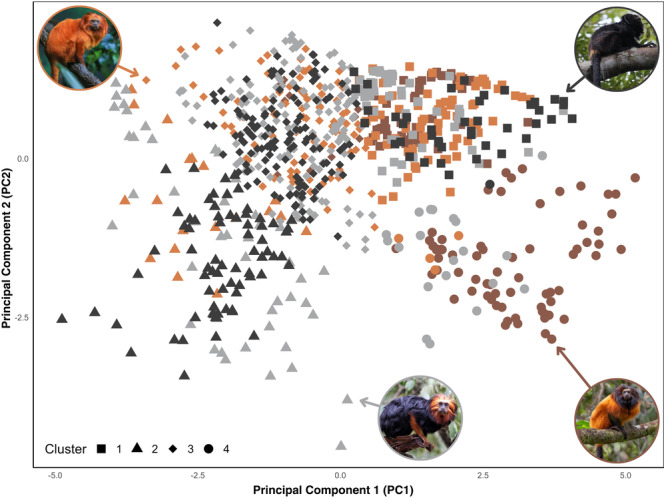
Scatter plot of principal component analysis (PCA) showing the four lion tamarin species, including *Leontopithecus rosalia* (orange), *Leontopithecus caissara* (brown), *Leontopithecus chrysomelas* (gray), and *Leontopithecus chrysopygus* (black), based on the acoustic parameters of seven different vocalizations, including long calls, whines, trills, rasps, clucks, tsicks, and peeps. The points represent the clusters obtained from cluster analysis, with each symbol (circle, triangle, square, and diamond) corresponding to a distinct cluster. PC1 and PC2 explain 66.26% and 27.43% of the variance, respectively. Photos: *L. chrysopygus*: Maria Carolina R. Manzano; *L. caissara*: Paulo Dimas D. Mascaretti Jr; *L. rosalia* and *L. chrysomelas* are sourced from Canva.

**Figure 3:**
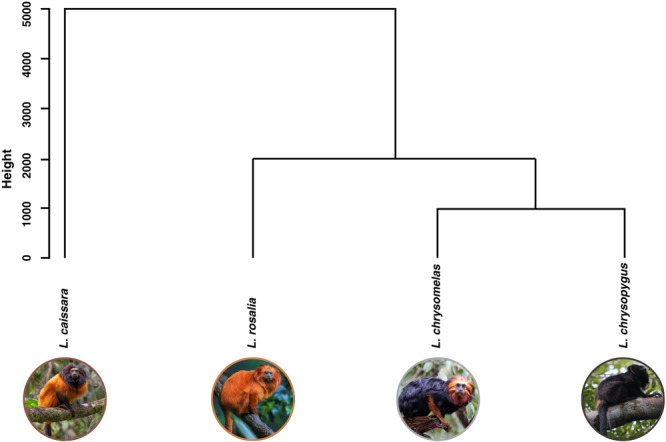
Dendrogram of hierarchical cluster analysis illustrating the relationships among the black‐faced lion tamarin (*Leontopithecus caissara*), golden lion tamarin (*Leontopithecus rosalia*), black lion tamarin (*Leontopithecus chrysopygus*), and golden‐headed lion tamarin (*Leontopithecus chrysomelas*), based on acoustic parameters of seven different vocalizations, including long calls, whines, trills, rasps, clucks, tsicks, and peeps. The vertical axis (“Height”) represents cluster distance. Photos: *L. chrysopygus*: Maria Carolina R. Manzano; *L. caissara*: Paulo Dimas D. Mascaretti Jr; *L. rosalia* and *L. chrysomelas* are sourced from Canva.

The PCA revealed that the first principal component (PC1) accounted for 66.26% of the total variance. In comparison, the second principal component (PC2) contributed an additional 27.43%, bringing the cumulative variance explained by the first two components to 93.69% (Figure 2). Together, these components provide a significant representation of the data structure. PC1 is primarily influenced by the frequency variables: frequency at 5% (−0.457), frequency at 95% (−0.482), peak frequency (−0.496), and center frequency (−0.53). In contrast, PC2 is predominantly represented by the frequency at 5% (0.407), frequency at 95% (−0.376), peak frequency (0.166), and bw90% (−0.809). Furthermore, cluster analyses revealed that the black lion tamarin (*L. chrysopygus*) is closest to the golden‐headed lion tamarin (*L. chrysomelas*), while it is furthest from the *L. caissara*, which is considered the most different species in terms of acoustic parameters (Figure 3).

## 4 Discussion

Our analysis reveals that while the four species of *Leontopithecus* share the seven types of vocalizations included in this study, as clucks, tsicks, trills, rasps, whines, peeps, and long calls, there are significant acoustic differences between them across the vocalizations. We demonstrate that spectral parameters, specifically frequency at 95%, peak frequency, center frequency, and BW90, are more relevant for distinguishing species than temporal parameters. The species *L. caissara* emerged as the most acoustically distinct, showing the highest number of significant paired comparisons (15), particularly in long calls and in four of the six other vocalization types. In contrast, *L. rosalia* and *L. chrysopygus* exhibited the most similar vocal profiles, followed by *L. chrysomelas* (13 significant comparisons) and *L. chrysopygus* (12). These findings detail the bioacoustics of the genus and highlight the potential of vocalizations, especially long calls, as tools for species identification.

It is important to note that some patterns may be influenced by the number of vocalizations analyzed, particularly the smaller sample size for *L. caissara*. Moreover, the overlap observed in the PCA may reflect multiple, non‐exclusive processes, such as partial acoustic convergence driven by similar ecological or social pressures, or shared environmental and geographical influences. Because all four species inhabit forested habitats, the acoustic properties of these environments may act as an environmental filter, favoring signals that propagate efficiently.

Our acoustic data showed that *L. chrysopygus* and *L. rosalia* would be the most similar species when we consider the long call, while *L. chrysopygus* and *L. caissara* are the species that show the most differences. While the vocal similarity between *L. chrysopygus* and *L. rosalia* is consistent with the mitochondrial DNA phylogeny that groups them as sister species (Perez‐Sweeney et al. 2008), current studies indicate closer proximity of *L. chrysopygus* and *L. caissara* (P. Freitas, personal communication). This phylogenetic hypothesis implies that the differences in vocalizations between these two species could not be a product of phylogenetic separation. Instead, they may result from acoustic divergence driven by adaptation to their different habitats.

Field studies on callitrichids indicate greater vocal repertoire differences in species that are geographically proximate. Mendes et al. (2009) observed differences in long‐distance calls between *Callithrix geoffroyi* and *C. kuhli*, species that inhabit similar environments and are geographically close. In our case, this would apply to what was observed by *L. caissara* and *L. chrysopygus*. *L. caissara* is distributed along the states of Paraná and São Paulo in low‐altitude tropical rainforest areas near the coast, whereas *L. chrysopygus* is found in the inland seasonal semideciduous forest of the state of São Paulo. Although these species do not share overlapping distributional areas, they are geographically closest (Garbino et al. 2016), yet exhibit pronounced divergence in their vocalizations. This scenario could highlight the role of environmental selection in shaping vocalizations. More genetic studies, as well as integrative phylogenies, with molecular, morphological, and behavioral data—including these vocalization parameters (Garbino and Martins‐Junior 2018)—would contribute to enhancing our understanding of the phylogenetic relationships of species and how this reflects on specific vocalizations.

In conclusion, our study provides the first comparative analysis of the seven shared vocalization types across all four species of *Leontopithecus*, establishing an acoustic baseline and confirming the importance of spectral parameters for species differentiation. The distinctiveness of species and the relationship between vocalizations, phylogeny, and geography highlight possibilities for future research. Future studies should focus on: (1) quantifying how the identified spectral parameters vary with individual and social context; (2) developing automated classifiers for species identification based on vocalization parameters; and (3) examining how the acoustic divergence we documented relates to phylogeny and environmental and landscape variables. All lion tamarin species are endangered, and our results can contribute to understanding their ecology and support their conservation and management. Based on our findings on differences in vocalizations between species, we can understand in future work how vocalizations vary according to the environment and explore individual variations in vocalizations to estimate population density and monitor future demographic changes using noninvasive methods.

## Author Contributions

**Maria Carolina Rodella Manzano:** conceptualization (lead), data curation (lead), formal analysis (lead), funding acquisition (lead), investigation (lead), methodology (lead), project administration (lead), software (lead), validation (lead), visualization (lead), writing–original draft (lead), writing–review and editing (lead). **Ricardo J. Sawaya:** conceptualization (supporting), data curation (supporting), formal analysis (supporting), investigation (equal), methodology (equal), project administration (supporting), supervision (equal), visualization (supporting), writing–original draft (equal), writing–review and editing (equal). **Gabriela Cabral Rezende:** conceptualization (equal), data curation (equal), formal analysis (equal), investigation (equal), methodology (equal), project administration (equal), software (supporting), supervision (equal), visualization (equal), writing–original draft (equal), writing–review and editing (equal). **Maria Luisa da Silva:** conceptualization (equal), data curation (supporting), formal analysis (supporting), investigation (equal), methodology (equal), project administration (supporting), supervision (lead), visualization (equal), writing–original draft (equal), writing–review and editing (equal).

## Conflicts of Interest

The authors declare no conflicts of interest.

## Supporting information

Manzanoetal AJP 2025 SMI.

## Data Availability

All sound recordings used are publicly available in the Macaulay Library (Cornell Lab of Ornithology) and the Fonoteca Neotropical Jacques Vielliard (FNJV, UNICAMP). The data used were provided by Museu de Diversidade Biológica (MDBio) Audiovisual Collection, in the University of Campinas (UNICAMP, São Paulo, Brazil), integrated by the Fonoteca Neotropical Jacques Vielliard (FNJV), and by Macaulay Library at the Cornell Lab of Ornithology. The papers selected for the literature review are provided in Supporting Information SI.
